# Exercise-induced AMPK and pyruvate dehydrogenase regulation is maintained during short-term low-grade inflammation

**DOI:** 10.1007/s00424-014-1499-x

**Published:** 2014-04-02

**Authors:** Rasmus Sjørup Biensø, Jesper Olesen, Line van Hauen, Simon Meinertz, Jens Frey Halling, Lasse Gliemann, Peter Plomgaard, Henriette Pilegaard

**Affiliations:** 1 Centre of Inflammation and Metabolism, The Center for Physical Activity Research and August Krogh Centre, Department of Biology, University of Copenhagen, Universitesparken 13, 2100 Copenhagen Ø, Denmark; 2 Department of Nutrition Sport and Exercise, University of Copenhagen, Copenhagen, Denmark; 3 Centre of Inflammation and Metabolism and The Centre for Physical Activity Research, Department of Clinical Biochemistry, Rigshospitalet, Copenhagen, Denmark

**Keywords:** LPS, TNFα, Skeletal muscle, Substrate oxidation, Human, AMPK, Exercise

## Abstract

The aim of the present study was to examine the effect of lipopolysaccharide (LPS)-induced inflammation on AMP-activated protein kinase (AMPK) and pyruvate dehydrogenase (PDH) regulation in human skeletal muscle at rest and during exercise. Nine young healthy physically inactive male subjects completed two trials. In an LPS trial, the subjects received a single LPS injection (0.3 ng/kg body weight) and blood samples and vastus lateralis muscle biopsies were obtained before and 2 h after the LPS injection and immediately after a 10-min one-legged knee extensor exercise bout performed approximately 2½ h after the LPS injection. The exercise bout with muscle samples obtained before and immediately after was repeated in a control trial without LPS injection. The plasma tumor necrosis factor α concentration increased 17-fold 2 h after LPS relative to before. Muscle lactate and muscle glycogen were unchanged from before to 2 h after LPS and exercise increased muscle lactate and decreased muscle glycogen in the control (*P* < 0.05) and the LPS (0.05 ≤ *P* < 0.1) trial with no differences between the trials. AMPK, acetyl-CoA carboxylase (ACC) and PDH phosphorylation as well as PDHa activity were unaffected 2 h after LPS relative to before. Exercise decreased (*P* < 0.05) PDH and increased (*P* < 0.05) AMPK and ACC phosphorylation as well as increased (*P* < 0.05) PDHa activity similarly in the LPS and control trial. In conclusion, LPS-induced inflammation does not affect resting or exercise-induced AMPK and PDH regulation in human skeletal muscle. This suggests that metabolic flexibility during exercise is maintained during short-term low-grade inflammation in humans.

## Introduction

Skeletal muscle has an extraordinary ability to regulate substrate choice and utilization according to availability [11, 32]. The exercise-induced enhancement of glucose and fat oxidation in skeletal muscle ensures ATP production for muscle contractions, and the interaction between fatty acids and glucose regulates the relative fatty acid and glucose oxidation and contributes to efficient substrate utilization [32]. Regulation of substrate choice and substrate utilization may however be influenced by metabolic changes and contribute to metabolic dysfunction. For example, chronically elevated plasma free fatty acid (FFA) levels will inhibit glucose oxidation and elevated plasma glucose may inhibit fat oxidation in skeletal muscle [19, 23, 27, 32]. Similarly, metabolically related diseases are often associated with low-grade inflammation characterized by chronically elevated levels of circulating cytokines [25] like the pro-inflammatory cytokine tumor necrosis factor (TNF)α. Previous studies have linked TNFα to insulin resistance in rat and human skeletal muscle [17, 30], as well as indicated TNFα-mediated effects on substrate utilization [35, 40, 47].

The pyruvate dehydrogenase (PDH) complex has a key position in the regulation of substrate choice as it catalyzes the decarboxylation of pyruvate to acetyl CoA, which represents the entry of carbohydrate-derived substrate into the mitochondria for oxidation [33]. In accordance, exercise has been shown to induce a rapid increase in the activity of PDH in the active form (PDHa) in human skeletal muscle [18] concomitant with increased glucose oxidation [31]. Furthermore, elevated plasma FFA has been shown to be associated with a reduced exercise-induced increase in PDHa activity in human skeletal muscle [20] supporting that PDH contributes to regulating substrate utilization during exercise as part of the interaction between fatty acids and carbohydrates [32, 33]. The PDHa activity is thought mainly to be regulated by phosphorylation of the PDH-E1α subunit [22, 28] determined by the activity of PDH kinases (PDK), which phosphorylate and inactivate the enzyme and PDH phosphatases (PDP), which dephosphorylate and activate PDH. Previous studies have shown that the PDK4 protein content is up-regulated in rat and/or human skeletal muscle by fasting and high-fat diet [26, 46] and this regulation has been suggested to contribute to the associated changes in PDHa activity [16]. Although the PDK4 protein content has been shown to be unaffected during even prolonged exercise, the PDK4 protein content has been demonstrated to be rapidly regulated in human skeletal muscle by carbohydrate availability [20] indicating a potential role of acute changes in PDK4 protein content in PDH regulation.

Several previous studies suggest that inflammation influences PDH regulation. Repeated *Escherichia coli* injections in rats have been shown to reduce the concentration of active PDH complex and increase PDK activity [39, 40]. Moreover, the observations that treatment with TNFα reduced PDH activity in rat cardiomyocytes [47] and human immune cells [36] as well as the finding that treatment with TNFα binding protein alleviated the effects of inflammation on PDH activity [40] indicate that TNFα influences PDH regulation. These findings are further supported by a study showing that lipopolysaccharide (LPS) infusion for 24 h decreased PDHa activity and increased PDK4 protein content in rat skeletal muscle [13]. On the other hand, local infusion of LPS or TNFα in one leg did not change resting PDH-E1α phosphorylation in human skeletal muscle [6, 9], which may suggest that the impact of inflammation on PDH regulation at rest depends on species, dose, or duration of the treatment.

The intracellular energy sensor, AMP-activated protein kinase (AMPK), is also a key factor in the regulation of substrate utilization. AMPK activity is increased both by AMP-mediated allosteric regulation and by phosphorylation [44] leading to stimulation of several downstream processes aiming at increasing ATP production. In accordance, exercise increases AMPK phosphorylation and activity in skeletal muscle leading to an enhancement of fat oxidation in skeletal muscle through AMPK-mediated phosphorylation and inactivation of acetyl-CoA carboxylase (ACC) [43]. As ACC catalyzes the production of malonyl CoA, an inactivation of ACC leads to reduced production of malonyl-CoA with concomitantly less inhibition of carnitine palmitoyltransferase I and an increased fatty acid oxidation [15]. AMPK has been suggested to have anti-inflammatory effects [14], and AMPK phosphorylation has been reported to increase in human skeletal muscle after an LPS injection, although a concomitant increase in ACC phosphorylation was not observed [3]. This may suggest that inflammation modifies AMPK-mediated intracellular signaling in resting skeletal muscle.

Although previous studies indicate that inflammation affects PDH and AMPK regulation, it is yet unresolved how inflammation influences exercise-induced AMPK and PDH regulation in human skeletal muscle. Therefore, the aim of the present study was to test the hypothesis that LPS-induced short-term inflammation impairs AMPK and PDH regulation in human skeletal muscle at rest and in response to an acute exercise bout.

## Methods

### Subjects

Nine physically inactive young healthy male subjects in the range from 20 to 26 years of age with an average body mass index of 25.6 ± 3.7 (mean ± SD) participated in the study. The subjects were physically active less than 1 h per week. Each subject underwent a health examination by a medical doctor. The subjects could participate in the study if the VO_2max_ was less than 45 ml min^−1^ kg^−1^ and was approved by the medical doctor. This fitness level was chosen to obtain healthy but untrained subjects to avoid any potential effects of training status on the effect of short-term inflammation on AMPK and PDH regulation. A group of endurance trained subjects also completed the experimental trials, but for another purpose than the present study and are therefore not include in the present study. The subjects were given both written and oral information about the study and the subjects gave their written consent to participate in the study. The study was performed according to the Declaration of Helsinki and approved by the Copenhagen and Frederiksberg Ethical committee in Denmark (H-1-2012-108).

### Pre-testing

The VO_2max_ was measured for each subject using an incremental ergometer bicycle test (Monarch Ergomedic 839E) for use as inclusion criteria. In addition, Watt_max_ was determined during an incremental one-legged knee extensor exercise test on a modified ergometer bicycle as previously described [29]. The Watt_max_ was used to determine the resistance during the experimental trials.

### Experimental protocol

The subjects completed a LPS trial and a control trial separated by a least 7 days. For both trials, the subjects were instructed to eat a carbohydrate rich meal 1 h before arriving to the laboratory.

#### LPS trial

After arriving to the laboratory, a catheter was placed in the femoral artery and in the femoral vein of one leg and a venflon (BD, East Rutherford, NJ, USA) was placed in an antecubital vein in the forearm. Blood samples were obtained from the femoral catheters and a muscle biopsy was obtained from the vastus lateralis muscle using the needle biopsy method [8] with suction. Insertions for biopsies were made under local anesthesia (Lidocaine, AstraZeneca, Södertälje, Sweden). A LPS solution (100 ng/ml) was either freshly prepared from a LPS stock (The Clinical Center, Critical Care Medicine Department, Bethesda, MD, USA) or used within a week from the preparation with storage at −20 °C. An intravenous injection of 0.3 ng/kg LPS was given through an arm venflon approximately 2.5 h after the subjects had eaten breakfast. Additional blood samples were obtained 30, 60, 90, and 120 min after LPS injection and additional muscle biopsies at 1 and 2 h after LPS was given. The 1-h biopsy, all venous blood samples and arterial blood samples obtained at 30, 60 and 90 min after the LPS injection are not used in the present study except the venous sample before LPS for comparison of the arterial and venous TNFα level. After the 2-h blood and tissue sampling, the subjects were transferred to a chair connected to a one-legged knee extensor ergometer bicycle with the back of the chair lying down. The right foot of the subject was tied to a rod connected to the crank set of the modified ergometer bicycle. The subjects first performed 1-min passive knee extensions to warm up the leg followed by 5-min exercise at 50 % of Watt_max_ and 5-min exercise at 60 % of Watt_max_. Additional blood samples were obtained 8 min into the exercise and an additional muscle biopsy was obtained from the vastus lateralis muscle of the working leg immediately as the exercise was terminated after 10 min of exercise.

Individual insertions were made for each biopsy. Visual fat, connective tissue, and blood were removed from the biopsies, which were quickly frozen in liquid nitrogen. The muscle biopsy piece used for PDHa activity was frozen in liquid nitrogen within ~15 s. The muscle biopsies were stored at −80 °C. The blood was collected in EDTA containing tubes, which were centrifuged and plasma was collected and stored at −80 °C.

Ear temperature, mean arterial blood pressure (MAP), and heart rate were recorded every 15 min until 3 h after the LPS injection. MAP monitoring continued until at least 4 h after the LPS injection to insure that normal blood pressure regulation was re-established.

#### Control trial

After arriving to the laboratory, a venflon was inserted in a vein in the forearm and a blood sample was obtained through the venflon (BD). In addition, a muscle biopsy was obtained from the vastus lateralis muscle as described above. The subjects were placed in the chair connected to the one-legged knee extensor ergometer bicycle with the back of the chair lying down. The right leg of the subject was tied to the modified ergometer bicycle and 1 min of passive knee extensions was performed followed by 5-min exercise at 50 % Watt_max_ and 5-min exercise at 60 % Watt_max_ as in the LPS trial. An additional blood sample was drawn after 8 min of exercise and an additional vastus lateralis muscle biopsy was obtained from the working leg immediately after 10 min of exercise. Blood and muscle biopsies were handled as described for the LPS trial above.

### Plasma analyses

#### Plasma glucose and insulin

The blood was analyzed immediately for blood glucose (ABL, Radiometer 725 series Acid–base Analyzer, Denmark) and insulin was measured at The Department of Clinical Biochemistry at Rigshospitalet, Copenhagen, Denmark.

#### Plasma TNFα

The plasma TNFα was determined using a MSD multi-spot 96 wells plate with pre-coated antibodies (MesoScaleDiscovery, Gaithersburg, ML, USA). The plates were measured on MSD Sector Image 2400 plate reader. The data were analyzed using the Discovery Workbench 3.0 (MSD). The results were converted to a concentration by use of a standard curve constructed from a serial dilution of recombinant TNFα run alongside on each plate.

### Muscle analyses

#### Dividing of muscle tissue

A piece of wet weight muscle tissue was chipped off and used for the PDHa activity analysis. An additional part of the muscle biopsies was freeze-dried for at least 48 h and these samples were dissected free from visual blood and connective tissue under the microscope. The freeze-dried muscle tissue was weighted out for the different analyses and stored at −80 °C.

#### Muscle glycogen

The muscle glycogen concentration was determined on freeze-dried muscle tissue as glycosyl units after acid hydrolysis as previously described [24].

#### Muscle lactate

PCA extract was made on freeze-dried muscle tissue as previously described [7]. Muscle lactate was measured using the auto-fluorescence ability of NADH as previously described [7].

#### SDS-PAGE and Western blotting

The freeze-dried muscle was homogenized using a Tissue LyserII (Qiagen, Hilden, Germany) and the homogenized muscle samples were made into lysates as previously described [38]. The protein concentration was determined with the bicinchoninic acid method (Pierce, ThermoScientific, Rockford, IL, USA) using BSA as a standard.

The lysate samples were loaded on hand casted gels (7.5–10 %). After the gel electrophoresis, the proteins were blotted from the gel to a PVDF membrane (Millipore, Bedford, USA), blocked in 3 % fish gelatin solution and incubated with antibodies. The protein content and phosphorylation level were determined using antibodies towards AMPK Thr^172^ phosphorylation (2535; Cell Signaling Technology, Berverly, MA, USA), ACC Ser^221^ phosphorylation (07–303; Millipore, Bedford, USA), PDH Ser^293^ (site1), PDH Ser^300^ (site 2), PDH Ser^295^ (site 4) phosphorylation, PDH-E1α protein, AMPKα2 protein, and PDK4 protein (provided by Graham Hardie, Dundee University, Dundee, UK). ACC protein content was detected using streptavidin–HRP (Dako, Glostrup, Denmark). The bands on the membranes were visualized with ECL reagent (Millipore, Billerica, MA, USA) in a digital image system (GE healthcare, München, DE).

#### PDHa activity

The PDHa activity was determined as previously described [10, 12, 31]). In brief, muscle homogenate was prepared on ice from wet weight muscle tissue using a glass homogenizer (Kontes, Vineland, NJ, USA). Pyruvate was converted to acetyl CoA followed by determination of the acetyl CoA content by use of a radioactive assay. The PDHa activity was determined as the rate of conversion of pyruvate to acetyl CoA and normalized to the total creatine content in each sample as previously described [34].

### Statistics

Values presented are means ± SE. A paired *t* test was used to test the effect of LPS at rest. A two-way ANOVA with repeated measures was used to evaluate the effect of LPS and exercise. The data were log transformed if normality or equal variance test failed. If a significant main effect was detected, the student Newman–Keuls test was used to locate differences. Differences are considered significant at *P* < 0.05 and a tendency is reported for 0.05 ≤ *P* < 0.1. Statistical calculations were performed using SigmaPlot 11.0 (Systat Software, Inc, San Jose, CA, USA).

## Results

### Physical parameters

The mean arterial pressure was stable around 85–90 mmHg and the ear temperature was unchanged around 37.0–37.6 °C. The heart rate increased (*P* < 0.05) from 63 ± 3 before LPS to 83 ± 4 beats/min 3 h after the LPS injection (Table 1).

**Table 1:** Physiological and plasma parameters in the LPS trial

	Pre	2 h LPS/pre exercise	3 h LPS
Temperature (°C)	37.4 ± 0.2	37.3 ± 0.1	37.6 ± 0.2
MAP (mmHg)	87.8 ± 2.9	88.0 ± 3.4	87.9 ± 3.6
Heart rate (BPM)	63 ± 3	68 ± 4	86 ± 4*
Arterial plasma glucose (mmol/l)	6.4 ± 0.6	5.0 ± 0.1*	4.9 ± 0.2
Arterial plasma insulin (pmol/l)	135.8 ± 18.2	65.3 ± 10.8*	69.3 ± 8.9

Ear temperature (°C), mean arterial pressure (MAP; mmHg), and heart rate (beats/min) before (pre-LPS) and 2 h (2 h LPS) and 3 h (3 h LPS) after a single LPS injection as well as arterial glucose (mmol/l) and arterial insulin (pmol/l) at pre-LPS, 2 h LPS corresponding to before exercise (pre-exercise) and at 8 min of exercise (exercise). The values are mean ± SE

*P < 0.05 (significantly different from pre-LPS)

### Plasma parameters

#### Plasma glucose and insulin

The arterial plasma glucose concentration decreased (*P* < 0.05) 2 h after LPS relative to before the LPS injection. Exercise did not change the plasma glucose concentration in the LPS trial (Table 1).

The arterial plasma insulin concentration decreased (*P* < 0.05) 2 h after LPS injection relative to before LPS. Exercise did not affect the plasma insulin level in the LPS trial (Table 1).

#### Plasma TNFα

Plasma TNFα was determined as a measure of the LPS-induced inflammation. The plasma TNFα concentration before LPS injection was similar in the femoral artery and femoral vein suggesting that arterial and venous levels can be compared at least in a non-inflammatory state. The arterial plasma TNFα concentration increased (*P* < 0.05) ~17-fold 2 h after LPS injection relative to before LPS. Exercise did not change the plasma TNFα concentration in either trial. The arterial plasma TNFα concentration was 13–15-fold higher (*P* < 0.05) in the LPS trial than the venous plasma TNFα in the control trial before and at the end of exercise (Table 2).

**Table 2:** Plasma TNFα concentrations

		Pre-LPS	2 h LPS/pre-exercise	Exercise
TNFα (ng/l)	Control	–	0.97 ± 0.1	1.0 ± 0.1
	LPS	0.90 ± 0.1	15.5 ± 1.6*#	13.6 ± 1.3*#

Arterial (LPS trial) and venous (control trial) plasma tumor necrosis factor (TNF)α (ng/l) concentration before a single LPS injection (pre-LPS) and 2 h after the LPS injection corresponding to before exercise (2 h LPS/pre-exercise) and 8 min into an one-legged knee extensor exercise bout (exercise). The values are mean ± SE

*P < 0.05 (significantly different from pre-LPS); #*P* < 0.05 (significantly different from control at the given time point)

### Muscle analyses

#### Muscle glycogen

The muscle glycogen concentration was unaffected from before LPS injection to 2 h after LPS. Exercise reduced (*P* < 0.05) muscle glycogen 27 % in the control trial and tended to reduce (0.05 < *P* < 0.1) muscle glycogen 6 % in the LPS trial. There was no significant difference in the muscle glycogen concentration between the two trials neither before nor after exercise (Table 3).

**Table 3:** Muscle glycogen and lactate concentrations

		Pre-LPS	2 h LPS/pre-exercise	Post-exercise
Glycogen (mmol kg^−1^ dw)	Control	–	365 ± 38	267 ± 44*
	LPS	329 ± 32	391 ± 49	367 ± 35*
Lactate (mmol kg^−1^ dw)	Control	–	9.7 ± 1.9	66.4 ± 15.6*
	LPS	10.9 ± 1.3	10.8 ± 1.4	53.2 ± 13.3**

Glycogen (mmol kg^−1^ dry weight) and lactate (mmol kg^−1^ dry weight) concentration in the vastus lateralis muscle before a single injection of LPS (pre-LPS), 2 h after the LPS injection corresponding to before exercise (2 h LPS/pre-exercise), and immediately after 10 min of one-legged knee extensor exercise (post-exercise). The values are mean ± SE

*P < 0.05 (significantly different from pre-LPS); **0.05 ≤ *P* < 0.1 (tends to be significantly different from pre-LPS)

#### Muscle lactate

Muscle lactate did not change from before the LPS injection to 2 h after LPS. Exercise increased (*P* < 0.05) muscle lactate 6.8-fold in the control trial and tended to increase (0.05 ≤ *P* < 0.1) muscle lactate 4.9-fold in the LPS trial with no difference between the two trials neither before exercise nor after exercise (Table 3).

#### AMPK and ACC phosphorylation

**Fig. 1:**
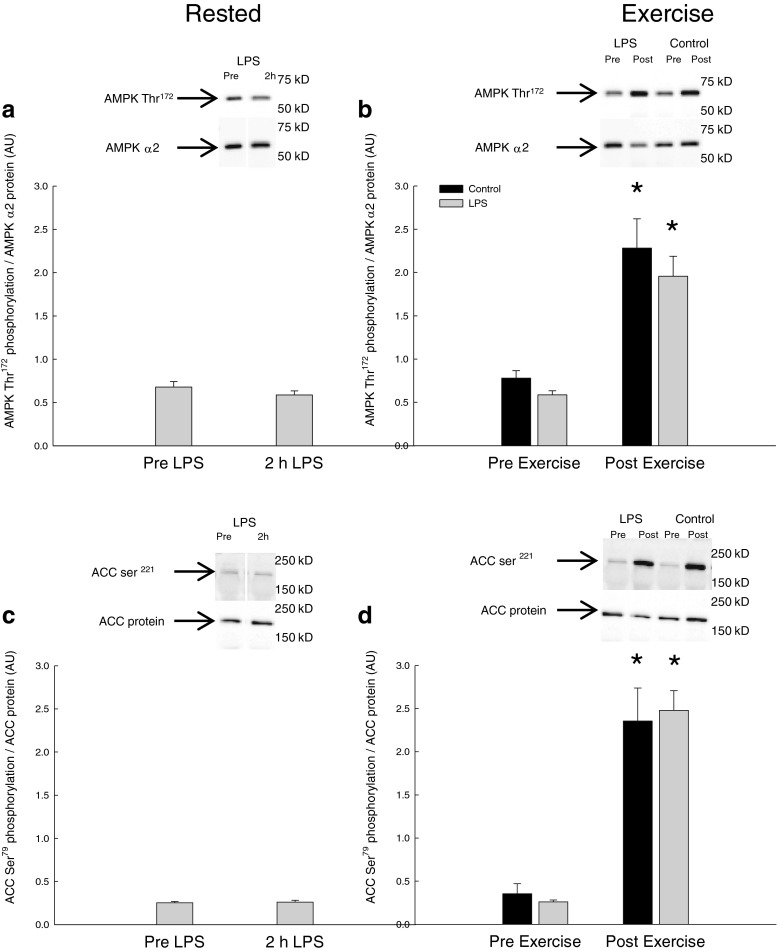
AMPK Thr^172^ phosphorylation normalized to AMPKα2 protein content **a** before (pre-LPS) and 2 h after LPS (2 h LPS) and **b** before (Pre-Exercise) and immediately after 10 min of one-legged knee extensor exercise (Post-Exercise). ACC Ser^221^ phosphorylation normalized to ACC2 protein content **c** pre-LPS and 2 h LPS as well as **d** Pre-Exercise and Post-Exercise. The results are presented as arbitrary units (AU). Values are mean ± SE. *P < 0.05, significantly different from pre-exercise

There was no difference in AMPK Thr^172^ and ACC Ser^221^ phosphorylation in skeletal muscle before and 2 h after LPS injection (Fig. 1a, c).

The exercise bout increased (*P* < 0.05) skeletal muscle AMPK phosphorylation ~3-fold and ACC phosphorylation 6–7-fold in the control and LPS trial with no difference in the responses between the trials. The AMPK and ACC phosphorylation levels were similar in the two trials both before and after exercise (Fig. 1b, d).

#### PDK4 protein

The PDK4 protein content in skeletal muscle was unchanged by LPS injection (pre-LPS, 0.98 ± 0.15; 2 h LPS, 1.11 ± 0.06) (Data not shown).

Exercise did not affect the PDK4 protein content in skeletal muscle and there was no difference between the control and LPS trial (control, pre-exercise, 1.29 ± 0.13; control, post-exercise, 1.12 ± 0.14; LPS, pre-exercise, 1.11 ± 0.06; LPS, post-exercise, 1.14 ± 0.11).

#### PDH-E1α phosphorylation

**Fig. 2:**
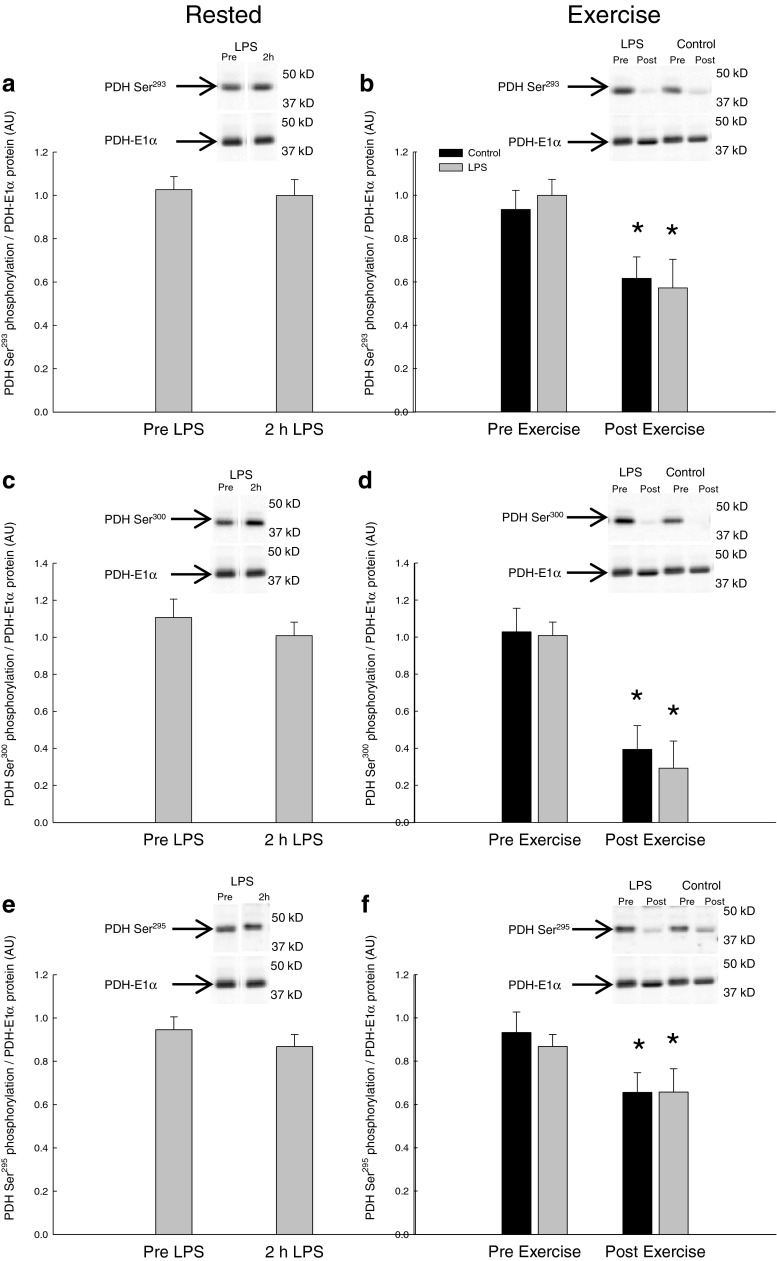
PDH Ser^293^ phosphorylation normalized to PDH-E1α protein content **a** before (pre-LPS) and 2 h after LPS (2 h LPS) and **b** before (Pre-Exercise) and immediately after 10 min of one-legged knee extensor exercise (Post-Exercise). PDH Ser^300^ phosphorylation normalized to PDH-E1α protein content **c** pre-LPS and 2 h LPS as well as **d** Pre-Exercise and Post-Exercise. PDH site Ser^295^ phosphorylation normalized to PDH-E1α protein content **e** pre-LPS and 2 h LPS as well as **f** Pre-Exercise and Post-Exercise. The results are presented as arbitrary units (AU). Values are mean ± SE. *P < 0.05, significantly different from Pre-Exercise

PDH-E1α phosphorylation at Ser^293^, Ser^300^, and Ser^295^ was unchanged 2 h after LPS injection relative to before (Fig. 2a, c, e).

Exercise decreased (*P* < 0.05) the PDH-E1α phosphorylation at site Ser^293^ 40–50 %, site^300^ 60–70 %, and site Ser^295^ 30–40 % in the control and LPS trial with no difference in the responses between the trials. The PDH-E1α phosphorylation level was for each of the three sites similar in the control and LPS trial both before and after exercise (Fig. 2b, d, f).

#### PDHa activity

**Fig. 3:**
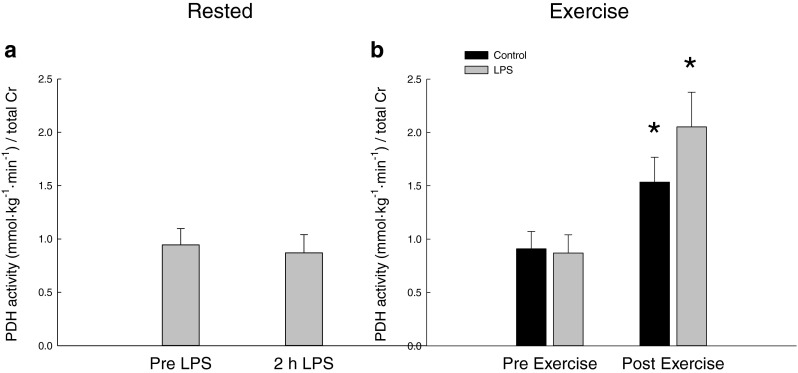
PDHa activity (mmol min^−1^ kg^−1^) **a** before (pre-LPS) and 2 h after LPS (2 h LPS) and **b** before (Pre-Exercise) and immediately after 10 min of one-legged knee extensor exercise (Post-Exercise). The PDHa activity is normalized to total creatine in the samples. Values are mean ± SE. *P < 0.05, significantly different from pre-exercise

The PDHa activity was unchanged 2 h after LPS injection relative to before LPS. Exercise increased (*P* < 0.05) the PDHa activity ~1.8-fold in the control trial and ~2.2-fold in the LPS trial with no difference in the response between the trials. The PDHa activity was similar in the two trials both before and after exercise (Fig. 3).

## Discussion

The main findings of the present study are that LPS-induced inflammation with elevated plasma TNFα concentration does not affect the exercise-induced AMPK and PDH regulation in human skeletal muscle. In addition, short-term inflammation does not affect AMPK and PDH regulation at rest.

The present human study used a single LPS injection to induce a controlled inflammation in young volunteers as a model for low-grade inflammation as frequently used [4, 5, 36, 37]. The present observation that the LPS injection increased the plasma TNFα concentration 17-fold to ~15 ng/l 2 h after the LPS injection is in accordance with several previous studies [4, 5, 37] and shows that the anticipated inflammatory state was obtained.

The observation that skeletal muscle PDHa activity and PDH phosphorylation did not change 2 h after LPS injection in the current study is different from previous observations in rats [13, 39, 40]. Hence 24 h of LPS infusion in rats has been shown to reduce the PDHa activity in skeletal muscle [13] and sepsis induced by repeated treatments with *E. coli* was associated with reduced concentration of active PDH in skeletal muscle [41]. The additional finding that treatment with TNFα binding protein prevented the *E. coli*-induced reduction in PDH activity suggested that TNFα is important in the LPS-induced effects on PDH during sepsis in rats [40]. Although the different observations may be due to species or model differences, it seems possible that the dose and the duration of treatment could be important. While a single injection of 0.3 ng kg^−1^ was used in the present study, effects on PDH were observed in rats with 24 h of LPS infusion resulting in an 8.9-fold increase in plasma TNFα [2, 13]. In addition, while the present study did not observe any change in muscle lactate 2 h after the LPS injection, a previous rat study using a high dose of LPS infusion (150 μg kg^−1^ h^−1^) reported that muscle lactate increased after 2 h of LPS treatment [2] indicating that the inflammation inhibited the PDH. Taken together, this may suggest that potential effects of LPS-induced inflammation on PDH regulation evolve later than 2 h after injection. But future experiments are needed to confirm this in humans.

Previous studies have also indicated a link between TNFα and AMPK, although different effects have been reported. The unchanged AMPK phosphorylation 2 h after the LPS injection in the present human study is hence not in accordance with a previous study showing that treatment of muscle cells with TNFα decreased the AMPK activity [35]. The current finding is in line with previous observations in humans, although AMPK phosphorylation increased in human skeletal muscle 4 h after a single LPS injection [3]. This may suggest that the different observations are due to model differences and the present findings do not oppose the possibility that LPS-induced inflammation in humans increases AMPK phosphorylation in skeletal muscle.

The present study is (to our knowledge) the first to examine the impact of LPS-induced inflammation on exercise-induced metabolic regulation in humans. The exercise-induced decrease in PDH phosphorylation and increase in AMPK and ACC phosphorylation as well as in PDHa activity in the control trial are as expected based on many similar previous studies [20, 21, 28, 45]. The present finding that the exercise-induced regulation of PDH, AMPK and ACC phosphorylation as well as PDHa activity was similar in the LPS trial, where TNFα was elevated, as in the control trial is however not as hypothesized. As PDH activation increases carbohydrate oxidation [31, 42] and AMPK-mediated ACC inactivation increases fat oxidation [15, 43], these observations suggest that human skeletal muscle maintains the ability to increase carbohydrate and fat oxidation in response to exercise despite the presence of short-term systemic inflammation. This conclusion is supported by the similar increase in the muscle lactate concentration in the two trials in the present study. In addition, it may be noted that while exercise induced a significant decrease in muscle glycogen and increase in muscle lactate in the control trials, these changes only tended to be significant in the LPS trial. This is opposite of expected based on previous animal LPS models indicating that inflammation results in elevated lactate production [2, 13]. However, the lower statistical strength of the muscle glycogen and lactate changes in the LPS trial does not change the conclusion that short-term inflammation does not affect exercise-induced PDH and AMPK regulation in human skeletal muscle, but may suggest that inflammation affects the utilization of muscle glycogen. But this remains to be elucidated.

The lack of effect of inflammation on exercise-induced PDH and AMPK regulation suggests that exercise-induced metabolic flexibility is maintained during short-term inflammation, which is in contrast to previous studies showing that elevated systemic TNFα levels [30] and a single LPS injection [1] induce whole-body insulin resistance in humans. These findings may suggest that insulin signaling and exercise-induced metabolic regulation are affected differently by inflammation. However, it is important to note that the lack of effect on the exercise-induced responses was observed 2½ h after LPS injection, while the LPS-induced lowering of insulin sensitivity was demonstrated 420 min after LPS was injected. On the other hand, the previously reported TNFα-induced insulin resistance was observed within 2 h of TNFα infusion with similar plasma levels of TNFα [30] as in the present study, while the previous LPS study resulted in more than 50-fold higher plasma TNFα concentration [1] than in the present and previous TNFα study [30]. Although the TNFα infusion did result in a constant elevation in plasma TNFα, while the present study induced a gradual increase in plasma TNFα, these considerations do support a different impact of TNFα on insulin-mediated glucose uptake than on exercise-induced AMPK and PDH regulation in human skeletal muscle.

As the purpose of the present study was to examine the potential effect of inflammation with elevated plasma TNFα, the exercise bout was placed at the time point where the plasma TNFα concentration was expected to peak. In accordance, the observation that plasma TNFα was at a similar level (15-fold elevated) and not further increased at the sampling time point during exercise (approximately 2½ h after LPS injection) relative to 2 h after LPS is in line with previous studies showing that plasma TNFα peaks at approximately 2 h after a single LPS injection [1, 3, 5, 37]. However, it is certainly possible that more long-term inflammation with more sustained elevation in plasma TNFα will affect the exercise-induced AMPK and PDH regulation in skeletal muscle, but this remains to be determined.

As previous studies examining the impact of LPS-induced inflammation and TNFα on PDH regulation have observed that a down-regulation of the PDHa activity was associated with increased PDK activity [39, 40] and PDK4 protein content [13], it may be that inflammation-induced effects on PDH regulation require changes in PDK4 expression, which may be obtained with high treatment dose and/or duration. The present observation that the lack of effect of inflammation on PDH regulation was associated with unchanged PDK4 protein content in skeletal muscle does support the possibility that changes in PDK4 protein underlie part of the effect of inflammation on PDH.

In conclusion, a single LPS injection resulting in 17-fold increase in plasma TNFα concentration did not change AMPK and PDH phosphorylation and/or activity in human skeletal muscle at rest and did not affect exercise-induced AMPK and PDH regulation in human skeletal muscle. This suggests that the ability of skeletal muscle to increase glucose and fat oxidation during exercise is maintained during short-term inflammation with elevated plasma TNFα levels. Hence, short-term low-grade inflammation does not seem to elicit metabolic inflexibility during exercise in humans as has been reported for insulin-stimulated glucose uptake at rest.
